# Copy number variations exploration of multiple genes in Graves’ disease

**DOI:** 10.1097/MD.0000000000005866

**Published:** 2017-01-27

**Authors:** Rong-hua Song, Xiao-qing Shao, Ling Li, Wen Wang, Jin-an Zhang

**Affiliations:** Department of Endocrinology, Jinshan Hospital of Fudan University, Jinshan District, Shanghai, China.

**Keywords:** copy number variation, Graves’ disease, microarray, single nucleotide polymorphism

## Abstract

**Background:**

Few previous published papers reported copy number variations of genes could affect the predisposition of Graves’ disease (GD). Herein, the aim of this study was to explore the association between copy number variations (CNV) profile and GD.

**Methods:**

The preliminary copy number microarray used to screen copy number variant genes was performed in 6 GD patients. Five CNV candidate genes (CFH, CFHR1, KIAA0125, UGT2B15, and UGT2B17) were then validated in an independent set of samples (50 GD patients and 50 matched healthy ones) by the Accucopy assay method. The CNV of the other 2 genes TRY6 and CCL3L1 was investigated in 144 GD patients and 144 healthy volunteers by the definitive genotyping technique using the Taqman quantitative polymerase-chain-reaction (Taqman qPCR). TRY6 gene-associated single nucleotide polymorphism (SNP), rs13230029, was genotyped by the PCR-ligase detection reaction (LDR) in 675 GD patients and 898 healthy controls.

**Results:**

There were no correlation of the gene copy number (GCN) of CFH, CFHR1, KIAA0125, UGT2B15, and UGT2B17 with GD. In comparison with that of controls, the GCN distribution of TRY6 and CCL3L1 in GD patients did not show significantly differ (*P* > 0.05). Furthermore, TRY6-related polymorphism (rs13230029) showed no difference between GD patients and controls. No correlation was found between CNV or SNP genotype and clinical phenotypes. Generally, there were no link of the copy numbers of several genes, including CFH, CFHR1, KIAA0125, UGT2B15, UGT2B17, TRY6, and CCL3L1 to GD.

**Conclusion:**

Our results clearly indicated that the copy number variations of multiple genes, namely CFH, CFHR1, KIAA0125, UGT2B15, UGT2B17, TRY6, and CCL3L1, were not associated with the development of GD.

## 1 Introduction

Graves’ disease (GD), one clinical subtype of autoimmune thyroid diseases (AITDs), is a kind of complex and thyroid-specific autoimmune disease with up to 5% in incidence.^[1]^ GD is characterized by the presence of hyperthyroidism and circulating thyroid stimulating hormone receptor antibody (TRAb). The disease predominantly affects women of child-bearing age. Likewise, GD has a high degree of familial aggregation and genetic heredity.^[2]^ Given the exact etiology of GD is yet to be defined, a large body of literature supports pivotal roles of the inherent genetic factors, environmental triggers, and their interaction.^[3]^ In addition, although under intense investigations, the genetic basis of human GD is still not well understood. Most genetic studies so far have focused on disease-association of GD with common genetic variation such as single-nucleotide polymorphism (SNP), but it has recently become apparent that copy-number variant (CNV) in immune genes is also a vital source of genomic diversity contributing to susceptibility to disease development as well.^[4,5]^

New high-throughput approaches such as microarray-based comparative genome hybridization (CGH) or CNV microarray, as an emerging and efficient molecular genetics technology, can simultaneously screen multiple CNV genes and areas in the whole genome on one specimen. In the present project, the CNV microarray was utilized to screen the CNV profile in GD. Then, we tried to further confirm the candidate CNV genes including CFH, CFHR1, KIAA0125, UGT2B15, and UGT2B17 in a larger size of samples (50 GD cases and 50 healthy ones). We also chose 2 immune-related genes, chemokine ligand 3-like 1 (CCL3L1), and trypsinogen C (TRY6), for in-depth verification on their copy numbers in GD. CCL3L1 is a variable copy-number gene which encodes a protein binding to several pro-inflammatory cytokine receptors including chemokine receptor 5 (CCR5). There is increasing evidence that CCL3L1 copy-number influences the susceptibility to rheumatoid arthritis (RA),^[6]^ type 1 diabetes (T1D),^[6]^ and systemic lupus erythematosus (SLE).^[7]^ Because the correlations between CNV of this gene (CCL3L1) and other autoimmune diseases have been reported,^[6,7]^ we attempted to grope for the gene copy number of CCL3L1 in GD. TRY6, whose alias is PRSS3P2, is localized to the T cell receptor (TCR) beta locus on chromosome 7. Given TRY6 is a key factor for T-cell-immune and inflammatory responses^[8,9]^ and simultaneously the microarray showed TRY6 was with less copy numbers in GD, we intended to explore whether TRY6 CNV is associated with the pathology of GD.

## 2 Subjects and methods

### 2.1 Subjects

Here, all GD patients were enrolled from the Outpatient Clinic of Endocrinology of Jinshan Hospital, whereas ethnically and geographically matched healthy controls were recruited from the Healthy Check-Up Center of the same hospital. All the patients and controls were given written informed consent. And sample collection was supported by the Ethical Committee of Jinshan Hospital of Fudan University. Each participant, including patients and controls, was ethnic Chinese Han.

All GD patients were newly diagnosed and met the diagnosis criteria, including clinical manifestations and biochemical assessments of hyperthyroidism and the positive circulating TRAb, with or without diffuse goiter of the thyroid and positive antibody against thyroid peroxidase (TPOAb) or thyroglobulin (TgAb), which was clearly described in our published articles previously.^[10,11]^ The subjects without positive antibody against TPO or Tg, personal or family history of thyroid diseases, or any other diseases were recruited as controls. In this study, the immunochemiluminescence method with high specificity and sensitivity was used for detecting TPOAb, TgAb, and TRAb, and all of the kits were provided by the Roche Company, China.

In the first screening stage, 6 GD patients were used for the microarray detection. In the validation stage, another 50 GD patients and 50 healthy subjects were enrolled. Finally, we chose 2 genes for further CNV genotyping, 1 gene chosen from CNV microarray (TRY6), and the other reported by other studies on other autoimmune diseases (CCL3L1). In the next stages, additional 2 independent cohorts were collected. For CNV genotyping, 144 GD patients and 144 matched controls were enrolled from the same hospital. And for SNP genotyping, another cohort of 675 Chinese patients with GD and 898 unrelated healthy controls were recruited for analysis in this phase.

### 2.2 DNA sample collection

Blood samples were collected from all the participants and genomic DNA was extracted from 2 mL of ethylenediamine tetra-acetic acid (EDTA)-treated peripheral venous blood using a commercially available kit of RelaxGene Blood DNA System (Tiangen Biotech, Beijing, China), following the instructions of the kit. The concentration and purity (represented as A260/A280 ratio) of all DNA samples were measured by the Nano Drop 2000 Spectro-photometer (Thermo Scientific Company, USA). The DNA samples with great quality (high purity and concentration) were prepared for next CNV and SNP genotyping.

### 2.3 CNV screening

The Affymetrix CytoScan HD array with 900,000 probes was applied for screening CNV genes and there were normal genes copy numbers on the slides as controls. For microarray detection, the sample DNA was digested by restriction enzymes Nspl, amplified by the PCR method, labeled and further fragmented to obtain biotin labeled DNA using the Affymetrix Cytoscan HD array kit (Cat #901835, Affymetrix, Santa Clara, CA) following the manufacturer's instructions. The array hybridization was performed at 50°C in Hybridization Oven (Cat #00-0331-220V, Affymetrix, Santa Clara, CA). After 16 hours’ incubation, array slides were washed in Fluidics Station (Cat #00-0079, Affymetrix, Santa Clara, CA) according to the user manual. Then, slides were scanned with default settings by the GeneChip Scanner 3000 (Cat #00-00212, Affymetrix, Santa Clara, CA) and Command Console Software 3.1 (Affymetrix, Santa Clara, CA). Data were extracted and further analyzed with Affymetrix Chromosome Analysis Suite (Affymetrix, Santa Clara, CA).

### 2.4 CNV genotyping

To detect copy number of each genomic DNA (gDNA) sample, we used 2 methods. First, the Accucopy assay (a multiple competitive real-time PCR, Shanghai Tianhao Company, China) was used for detecting 5 genes, CFH, CFHR1, KIAA0125, UGT2B15, and UGT2B17, chosen from the array detection. Ten target genomic segments within the 5 genes (2 segments for each gene) were chosen for the PCR measurement. The reference genomic sequences were 2p, 10pL, 20q, and 16p. The forward and reverse primers of target genome segments are displayed in Table 1. Then, we used the quantitative polymerase chain reaction (qPCR) by TaqMan Copy Number Assays (Applied Biosystems of Lifetechnology, Foster City, CA) for CCL3L1, (Hs03198166_cn) and TRY6 (Hs03114102_cn), according to the manufacturer's protocol. qPCR was performed using the applied biosystems (ABI) 20 μL copy number quantitation reaction that contained 20 ng genomic DNA, FAM dye-labeled target gene probe, VIC-TAMRA dye-labeled reference gene (RNase part number 4403328) probe, and TaqMan Genotyping Master Mix (2 × , number 4371355). The amplification reaction was performed using the Applied Biosystems 7300 Realtime PCR machine using the following PCR procedure, DNA polymerase enzyme activation at 95°C for 10 minutes (min), followed by 40 cycles of these steps: denaturation at 95°C for 15 seconds (s) and annealing/extension at 60°C for 1 minute. Data were collected at the end of each cycle. All DNA samples were run in triduplicates to ensure accurate results. Subsequently, the SDS software 1.4 (ABI, CA) automatically excluded samples with large standard deviations based on a predesigned algorithm. The results were measured as the threshold cycle (CT), which means the cycle number of the PCR product crossing the threshold of detection. Then, the CopyCaller Software (Applied Biosystems by LifeTechnology, CA) was used to analyze copy numbers of the tested gene in the samples. The software is based on the △△CT method and obtained copy number values are as follows: CT values were imported into the program and normalized to the reference gene generating a △CT value; to obtain the △△CT value, since all the DNA samples had unknown copy numbers, the program found the △CT value obtained in the standard DNA sample (EpiTect Control DNA, number 59568, Qiagen Company) and assigned this △CT value as that of a sample with 2 copies. Then, this △CT value subtracted from the △CT values of each other samples, generating a new △△CT value. The △△CT value was then used to get the relative quantification of the gene analyzed using the formula 2^−△△CT^. Finally, the copy numbers of all DNA samples were calculated and subjected to further statistical analysis.

**Table 1:**
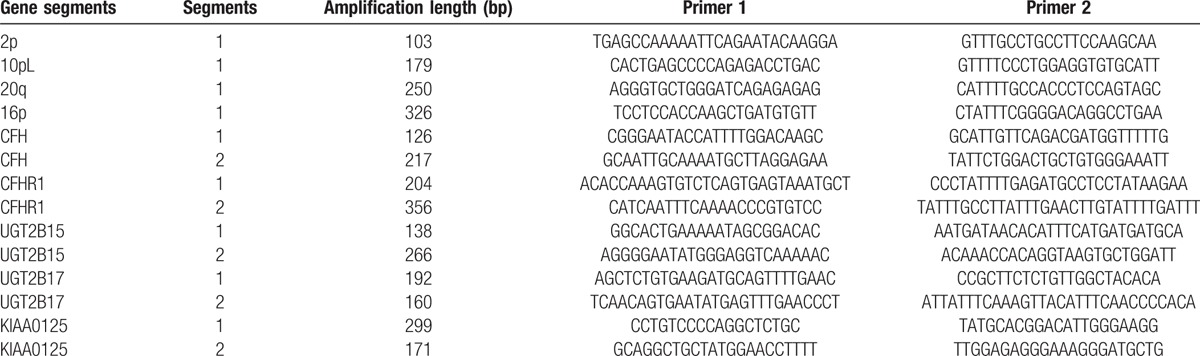
The primers of target genomic segments and reference genome sequences by the Accucopy assay.

### 2.5 SNP genotyping

All the SNP genotyping experiments were done using the PCR-ligase detection reaction (LDR). The target DNA sequences were amplified using a multiplex PCR method, and the PCR program (20 μL volume) was conducted as follows: 95°C 2 minutes, 94°C 30 s, 40 cycles (53°C 90 s, 65°C 30 s), 65°C 10 minutes. The ligation reaction for each sample was carried out in a final volume of 10 μL, containing 1 μL of 1× buffer, 4 μL of multi-PCR product, 1 μL of 2 pmol/μl probe mix, and 0.05 μL of 20U Taq DNA ligase (New England Biolabs). The LDR method was performed using 95°C 2 minutes, followed by 40 cycles of: 94°C for 15 seconds and 50°C for 25 seconds. The fluorescent products of LDR were differentiated by the software of Gene Amp PCR system 9600 (Norwalk, CT).

To ensure quality of the genotyping, we made encoded samples numbers, double positive controls (duplication of the same DNA samples) and negative controls (blank samples without DNA) in the process of SNP genotyping. Quality control analysis was also performed so that only SNPs and samples which had passed the 98% quality control threshold were subjected to further statistical analysis. All genotype call rates were manually recorded and conflicting results were liberally re-genotyped by sequencing. The information of primers and probes specific for the SNP (rs13230029) in the region of TRY6 is shown in Table 2.

**Table 2:**
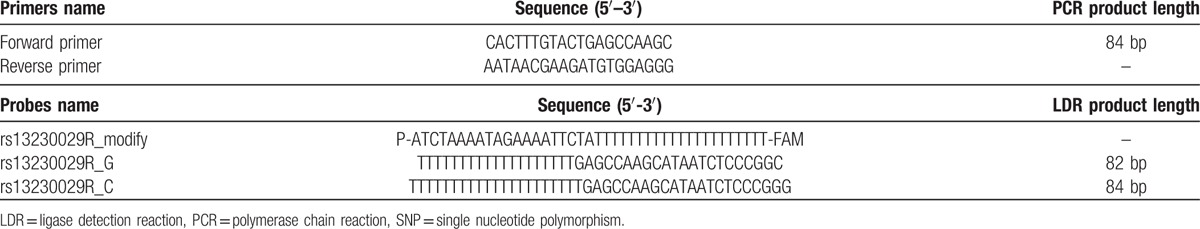
Probe and primer information for TRY6 SNP-genotyping using the PCR-LDR method.

### 2.6 Statistical analysis

The clinical data are described as mean ± standard deviation (M ± SD). Hardy–Weinberg equilibrium (HWE) analysis was conducted with Haploview software 4.2, using a threshold of 0.05, without correction for multiple testing. Comparison of cases and controls was made with allele frequency and copy number data using Pearson chi-square tests or Fisher's exact test on 2 × 2 contingency tables. Further, polymorphism genotype association was compared by chi-square tests on 2 × 3 contingency tables as well. Other statistical analyses were performed using a customized version of SPSS 17.0 (http://www-01.ibm.com/software/analytics/spss/). *P*-value of <0.05 was considered of statistical significance. Effect sizes, taken as odds ratio (*OR*), and 95% confidence interval (*95%CI*) assessed the association between each genotype and GD.

## 3 Results

### 3.1 Patients data

Demographic data and clinical characteristics of the 2-cohorts (GD patients and controls) are described in Table 3. In the first CNV microarray screening stage, we collected 6 GD patients (33.333% men and 66.667% females). In the validation study, we enrolled 50 GD patients (34.000% males and 66.000% females) and 50 controls (36.000% males and 64.000% females). Then, in subsequent study phase, our research investigated 144 GD patients (65.278% females and 34.722% males) and 144 controls (66.667% females and 33.333% males). In GD patients, the average onset age was 36.640 ± 14.797 years, 14 (9.722%) had ophthalmopathy and 32 (22.222%) individuals had family history. In the second SNP stage, we enrolled 675 GD patients (69.185% females and 30.815% males) and 898 controls (66.815% female sand 33.185% males). The average onset age of GD patients was 33.920 ± 14.516 years old, 120 (17.778%) had ophthalmopathy and 137 (20.296%) individuals were with family history.

**Table 3:**
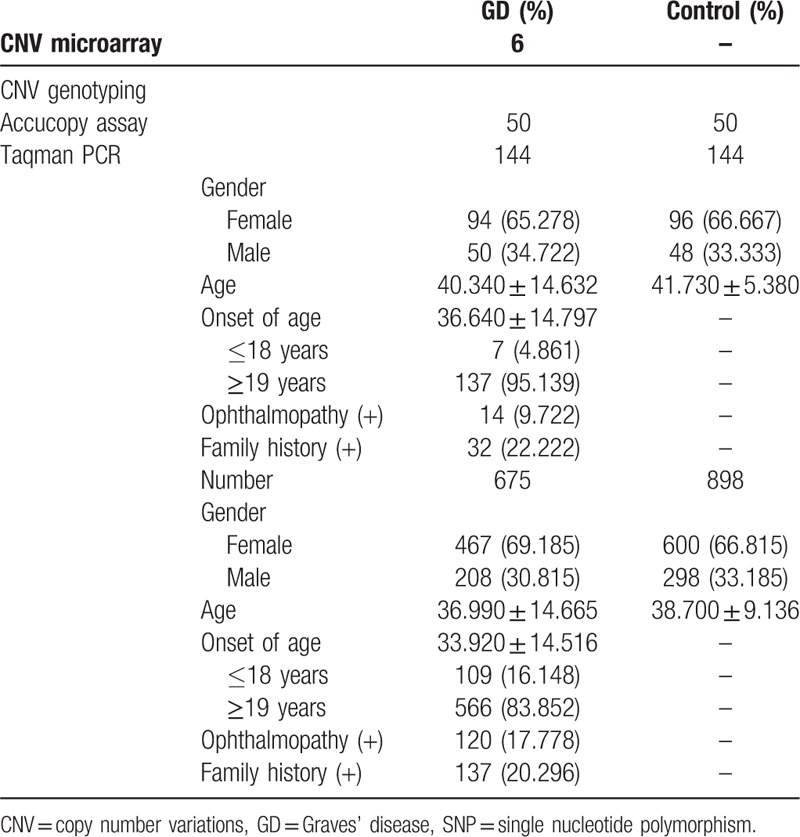
Demographic data and clinical characteristics from the 2 cohorts including GD patients and controls.

### 3.2 Gene copy number (GCN) array screening

Through CNV microarray detection, we found several CNV genes, which were shared by 6 GD patients, including some genes with copy number gain, such as KIAA0125, ADAM6, MFHAS1, IL3RA, SLC25A6, DHRSX, HDHD1, DMD, NROB1, FGF13, FAM70A, MID2, BRWD3, ACSL4, SLC6A14, SLC25A43, ZNF280C, CFH, and CFHR1, and some gene copy number loss genes, such as OR4P4, OR4S2, OR4C6, HEATR4, ACOT1, ANKRD36, UGT2B17, UGT2B15, TRY6, PRSS2, ASMTL, NLGN4X, DMD, RP2, IL1RAPL1, PCDH11X, KIAA2022, VAMP7, GPC3, ZDHHC9, and ASMTL-AS (as shown in Table 4).

**Table 4:**
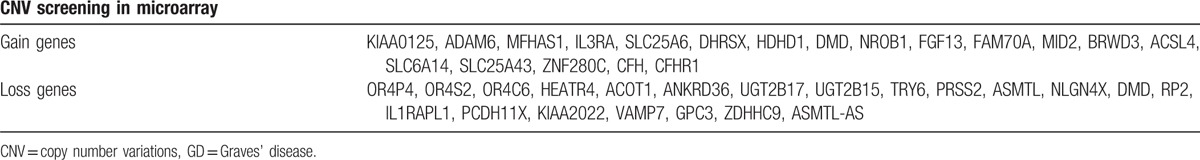
CNV profile in GD by microarray screening.

### 3.3 Gene copy number (GCN) association validation

#### 3.3.1 Accucopy assay validation

DNA copy number calculation in CFH showed no deletion or duplication in our GD cases. Association analysis of the copy number variations in CFHR1, KIAA0125, UGT2B15, and UGT2B17 did not find significant difference between cases and controls.

#### 3.3.2 CNV assay selection

To identify CNVs in the TRY6 and CCL3L1 genes, we searched the Database of Genomic Variants (DGV; http://projects.tcag.ca/variation) for cataloged CNVs. TRY6 has only one listed designed-probe in DGV and can be detected by an assay specific for this CNV. That is what we chose for detection. However, CCL3L1 has multiple cataloged probes in DGV, which overlap with and encompass the entire gene. Then, we selected one, which was reported by several CNV array previously, for further CNV genotyping.

#### 3.3.3 CNV analysis in the TRY6 gene

The gene copy number (GCN) of TRY6 was confirmed in our cohort. It varied from 0 to 3. 0 was the most common GCN count. In comparison with that of the controls, the GCN distribution of TRY6 in GD patients did not significantly differ (shown in Table 5).

**Table 5:**
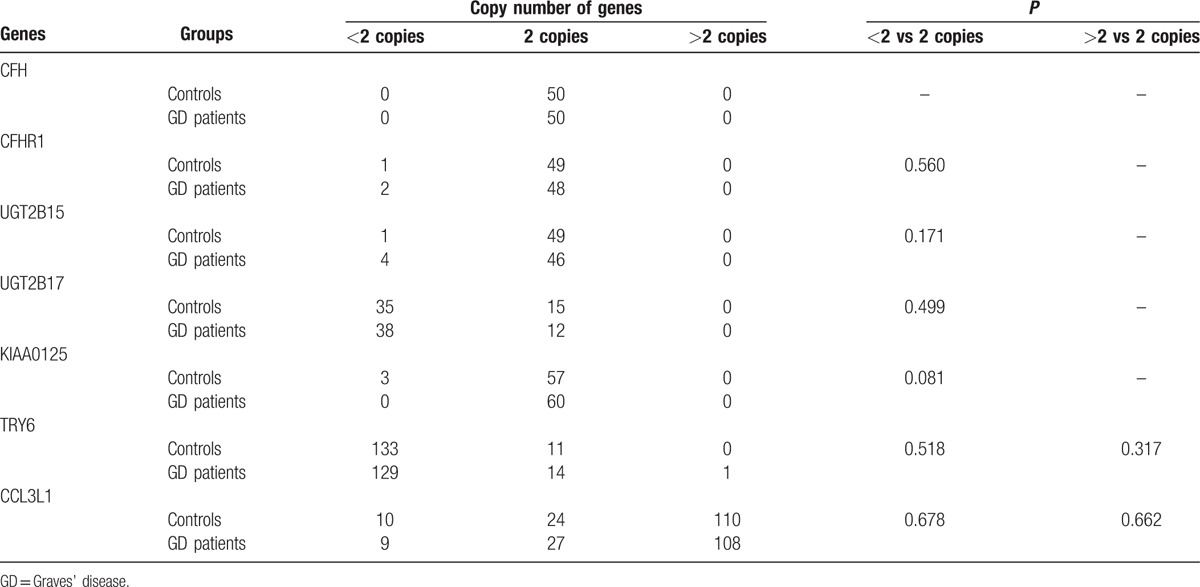
Comparison of the copy numbers frequencies in CFH, CFHR1, KIAA025, UGT2B15, UGT2B17, TRY6, and CCL3L1 between GD patients and controls.

#### 3.3.4 CNV analysis of the CCL3L1 gene

As shown in Table 5, there was indeed CCL3L1 CNV in GD patients and controls. That of CCL3L1 was from 1 to 5, and 5 copies was the most common. In comparison with that of controls, the CCL3L1 gene in GD patients did not show difference in GCN distribution (*P* > 0.05).

### 3.4 Correlation of CNV with clinical phenotype in GD patients

We further analyzed the correlation of the CNV distribution with GD patients with or without clinical phenotype (female or male, with or without family history, with or without ophthalmopathy, the age of onset ≤18 years old or ≥19 years old), but the results were all negative (*P* > 0.05, the data not shown).

### 3.5 Allelic and genotypic association of SNP

In our cohort, TRY6 CNV-associated SNP locus, rs13230029, was in Hardy–Weinberg equilibrium (*P*-value larger than 0.05). Allele G of rs13230029 in GD patients was up to 29.778%, higher than that in healthy subjects (29.399%), but without significant difference (*P* = 0.818). When the genotype distribution was further analyzed, the results displayed that GG genotype of TRY6 gene in the GD group was increased compared with that in the control group, but with no significant difference (displayed in Table 6). Similarly, we compared the allele and genotype distributions in clinical phenotype of GD patients (gender, onset of age, with or without ophthalmopathy, with or without family history), but no positive results were found (data not shown).

**Table 6:**
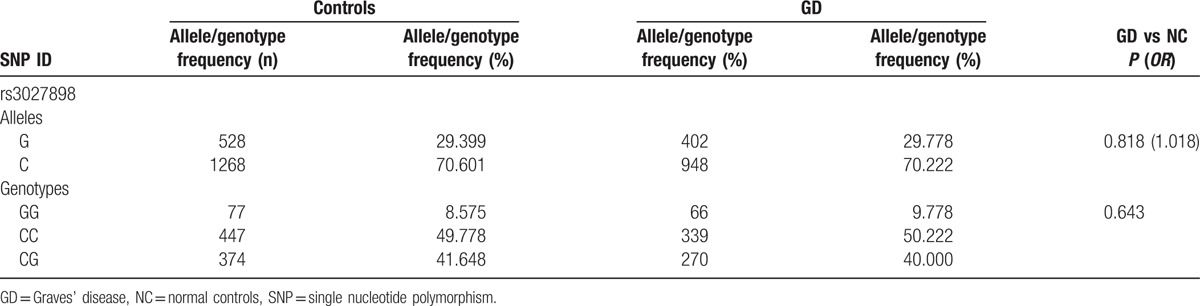
Allele and genotype frequencies of rs13230029 in GD patients and controls.

## 4 Discussion

Hitherto, significant progress has been made in understanding the role of genetic factors in the pathomechanism of GD.^[2]^ Recent data on the genetic susceptibility to GD reveal a novel mechanism. For instance, a large body of evidence show that SNPs of different genes are involved in the development of GD, such as the polymorphisms of IL-21/IL-21R,^[12]^ BANK1,^[13]^ STAT3,^[14]^ and TNFAIP3.^[15]^ Several researches have concluded that the varieties of DNA copy number, another essential subtype of genetic components, may influence the expression of genes and change the structure of genes, and therefore trigger the contribution to substantial phenotypic variation. Moreover, CNV in genomic regions harboring many dosage-sensitive genes probably cause or predispose to a variety of human genetic diseases. So far, increasing data have suggested that the CNVs of immune-genes may confer risk for various autoimmune diseases, with a manner similar to the well-documented associations between SNPs in immune-related genes and autoimmunity. For example, C4 CNVs were associated with SLE.^[16]^ In CD, HBD-2 showed fewer copy numbers,^[17]^ and CNVs of FCGR3B were linked to RA^[18]^ and SLE.^[19]^ However, few published papers reported whether copy number variations of genes could affect the predisposition of GD.^[4,5,20,21]^

Here, CNV microarray as a genome-wide analytical technique was applied by us to detect genomic CNV profile in GD, and we indeed found some copy number variant genes not only on enchromosomes but also on sex chromosomes. However, 5 genes including CFH, CFHR1, KIAA0125, UGT2B15, and UGT2B17, which were chosen from the CNV assay detection, showed no relation to GD in further validation procedure.

It was showed TRY6 with gene copy numbers loss in GD patients in our microarray screening and it was considered the pivotal roles of CCL3L1, TRY6 as vital effectors in immune-regulation, so our present study explored whether there is an association between copy number variation of these 2 immune-genes and the pathogenesis of GD. The copy number of CCL3L1 varies among individuals, with most subjects owning 1 to 5 copies in the diploid genome. It was reported that the low CCL3L1 copy number is an important risk factor for HIV infection, whereas high CNV of CCL3L1 is protective against the disease.^[22]^ A more recent publication reported the lower copy number (less than 2) of CCL3L1 increased the risk of SLE.^[7]^ And another study suggested that CCL3L1 CNV was associated with the susceptibility to RA and T1D, probably through enhancing inflammatory responses and increasing the chance of autoimmune diseases.^[6]^ Our present study showed there was indeed CCL3L1 CNV in GD patients and controls, from 1 copy to 5 copies. Nevertheless, the CNV distribution of CCL3L1 did not differ between the GD group and the control group. Further, we also investigated the correlation of the clinical subsets with the CNV distribution, and found no statistical significance in all case-only comparisons (cases with each clinical phenotype versus those without).

TRY6 is a member of a highly homologous serine protease family (PRSS). It is also a locus that appears to encode a protein trypsinogen and this gene is related to the T cell receptor (TCR). In recent years, TRY6 has been believed to be involved in immune and pro-inflammatory processes.^[8]^ Moreover, TRY6 mutation is associated with an inflammatory disease, pancreatitis.^[23]^ Rs13230029, a neighboring SNP in 100% LD with the TRY6 gene deletion is associated with breast cancer risk.^[24]^ In our previous microarray detection, there were less gene copies of TRY6 in GD patients. Nevertheless, in our present study, neither TRY6 CNV nor rs13230029 locus was found to be associated with GD. Furthermore, we analyzed the correlation of the clinical subsets with the TRY6 CNV and SNP allele frequencies, and no statistical significance was found in comparison with each clinical phenotype. Therefore, the TRY6 copy number and neighboring SNP both showed no link with GD, suggesting that TRY6 gene probably does not affect the susceptibility to GD.

Obviously, our research is only a preliminary and important exploration into copy number variation of immune-related genes, namely CFH, CFHR1, KIAA0125, UGT2B15, UGT2B17, CCL3L1, and TRY6, in GD. In spite of our negative results, there are investigations reported that copy number variants of C4,^[4]^ TSHR, and TLR7,^[5]^ but not FCGR3B,^[20]^ CD40 and CTLA4,^[24]^ confer risk to GD. Therefore, in the future more genetic, cellular and molecular experiments in depth are warranted to validate our results.

## 5 Conclusions

To summarize, our results for the first time indicated the copy number variations of genes, namely CFH, CFHR1, KIAA0125, UGT2B15, UGT2B17, TRY6, and CCL3L1, are not associated with the risk for GD.

## References

[1] Tomer Y Huber A The etiology of autoimmune thyroid disease: a story of genes and environment. J Autoimmun 2009;32:231–9. 19307103 10.1016/j.jaut.2009.02.007 PMC3561494

[2] Simmonds MJ GWAS in autoimmune thyroid disease: redefining our understanding of pathogenesis. Nat Rev Endocrinol 2013;9:277–87. 23529038 10.1038/nrendo.2013.56

[3] Weetman AP Autoimmune thyroid disease: propagation and progression. Eur J Endocrinol 2003;148:1–9. 12534350 10.1530/eje.0.1480001

[4] Liu YH Wan L Chang CT Association between copy number variation of complement component C4 and Graves’ disease. J Biomed Sci 2011;18:71. 21943165 10.1186/1423-0127-18-71 PMC3212822

[5] Liao WL Wan L Wang TY Association of TLR7 and TSHR copy number variation with Graves’ disease and Graves’ ophthalmopathy in Chinese population in Taiwan. BMC Ophthalmol 2014;14:15. 24517461 10.1186/1471-2415-14-15 PMC3929160

[6] McKinney C Merriman ME Chapman PT Evidence for an influence of chemokine ligand 3-like 1 (CCL3L1) gene copy number on susceptibility to rheumatoid arthritis. Ann Rheum Dis 2008;67:409–13. 17604289 10.1136/ard.2007.075028

[7] Mamtani M Rovin B Brey R CCL3L1 gene-containing segmental duplications and polymorphisms in CCR5 affect risk of systemic lupus erythaematosus. Ann Rheum Dis 2008;67:1076–83. 17971457 10.1136/ard.2007.078048 PMC3786698

[8] Rygiel AM Beer S Simon P Gene conversion between cationic trypsinogen (PRSS1) and the pseudogene trypsinogen 6 (PRSS3P2) in patients with chronic pancreatitis. Hum Mutat 2015;36:350–6. 25546417 10.1002/humu.22747 PMC4361298

[9] Rowen L Koop BF Hood L The complete 685-kilobase DNA sequence of the human beta T cell receptor locus. Science 1996;272:1755–62. 8650574 10.1126/science.272.5269.1755

[10] Song RH Yu ZY Qin Q Different levels of circulating Th22 cell and its related molecules in Graves’ disease and Hashimoto's thyroiditis. Int J Clin Exp Pathol 2014;7:4024–31. 25120780 PMC4129015

[11] Song RH Qin Q Yan N Variants in IRAK1-MECP2 region confer susceptibility to autoimmune thyroid diseases. Mol Cell Endocrinol 2015;399:244–9. 25458699 10.1016/j.mce.2014.10.013

[12] Zhang J Xiao WX Zhu YF Polymorphisms of interleukin-21 and interleukin-21-receptor genes confer risk for autoimmune thyroid diseases. BMC Endocr Disord 2013;13:26. 23889847 10.1186/1472-6823-13-26 PMC3766107

[13] Muhali FS Song RH Wang X Genetic variants of BANK1 gene in autoimmune thyroid diseases: a case-control association study. Exp Clin Endocrinol Diabetes 2013;121:556–60. 24127308 10.1055/s-0033-1348220

[14] Xiao L Muhali FS Cai TT Association of single-nucleotide polymorphisms in the STAT3 gene with autoimmune thyroid disease in Chinese individuals. Funct Integr Genomics 2013;13:455–61. 24081513 10.1007/s10142-013-0337-0 PMC3824579

[15] Song RH Yu ZY Wang Q Polymorphisms of the TNFAIP3 region and Graves’ disease. Autoimmunity 2014;47:459–65. 24798189 10.3109/08916934.2014.914504

[16] Yang Y Chung EK Wu YL Gene copy-number variation and associated polymorphisms of complement component C4 in human systemic lupus erythematosus (SLE): low copy number is a risk factor for and high copy number is a protective factor against SLE susceptibility in European Americans. Am J Hum Genet 2007;80:1037–54. 17503323 10.1086/518257 PMC1867093

[17] Fellermann K Stange DE Schaeffeler E A chromosome 8 gene-cluster polymorphism with low human beta-defensin 2 gene copy number predisposes to Crohn disease of the colon. Am J Hum Genet 2006;79:439–48. 16909382 10.1086/505915 PMC1559531

[18] Graf SW Lester S Nossent JC Low copy number of the FCGR3B gene and rheumatoid arthritis: a case-control study and meta-analysis. Arthritis Res Ther 2012;14:R28. 22309893 10.1186/ar3731 PMC3392823

[19] Molokhia M Fanciulli M Petretto E FCGR3B copy number variation is associated with systemic lupus erythematosus risk in Afro-Caribbeans. Rheumatology (Oxford) 2011;50:1206–10. 21296850 10.1093/rheumatology/keq456 PMC3670581

[20] Fanciulli M Norsworthy PJ Petretto E FCGR3B copy number variation is associated with susceptibility to systemic, but not organ-specific, autoimmunity. Nat Genet 2007;39:721–3. 17529978 10.1038/ng2046 PMC2742197

[21] Huber AK Concepcion ES Gandhi A Analysis of immune regulatory genes’ copy number variants in Graves’ disease. Thyroid 2011;21:69–74. 21054240 10.1089/thy.2010.0262 PMC3012451

[22] Shao W Tang J Song W CCL3L1 and CCL4L1: variable gene copy number in adolescents with and without human immunodeficiency virus type 1 (HIV-1) infection. Genes Immun 2007;8:224–31. 17330138 10.1038/sj.gene.6364378

[23] Chen JM Ferec C Genes, cloned cDNAs, and proteins of human trypsinogens and pancreatitis-associated cationic trypsinogen mutations. Pancreas 2000;21:57–62. 10881933 10.1097/00006676-200007000-00052

[24] Wagner K Grzybowska E Butkiewicz D High-throughput genotyping of a common deletion polymorphism disrupting the TRY6 gene and its association with breast cancer risk. BMC Genet 2007;8:41. 17598925 10.1186/1471-2156-8-41 PMC1925117

